# Culex quinquefasciatus (Diptera: Culicidae) survivorship following the ingestion of bird blood infected with Haemoproteus sp. parasites

**DOI:** 10.1007/s00436-021-07196-7

**Published:** 2021-06-10

**Authors:** Dayvion R. Adams, Andrew J. Golnar, Sarah A. Hamer, Michel A. Slotman, Gabriel L. Hamer

**Affiliations:** 1 Department of Entomology, Texas A&M University, 2475 TAMU, College Station, TX 77843 USA; 2 Schubot Center for Avian Health, Department of Veterinary Pathobiology, Texas A&M University, 4467 TAMU, College Station, TX 77843 USA; 3 Present Address: United States Department of Agriculture, Animal and Plant Health Inspection Service, 4101 LaPorte Avenue, Fort Collins, CO 80521 USA; 4 Department of Veterinary Integrative Bioscience, Texas A&M University, 4458 TAMU, College Station, TX 77843 USA

**Keywords:** Survivorship, Vectorial capacity, Mosquito, Birds, Pathogen

## Abstract

Arthropod vectors are frequently exposed to a diverse assemblage of parasites, but the consequence of these infections on their biology and behavior are poorly understood. We experimentally evaluated whether the ingestion of a common protozoan parasite of avian hosts (*Haemoproteus* spp.; Haemosporida: Haemoproteidae) impacted the survivorship of *Culex quinquefasciatus* (Say) (Diptera: Culicidae). Blood was collected from wild northern cardinals (*Cardinalis cardinalis*) in College Station, Texas, and screened for the presence of *Haemoproteus* spp. parasites using microscopic and molecular methods. Experimental groups of *Cx. quinquefasciatus* mosquitoes were offered *Haemoproteus*-positive cardinal blood through an artificial feeding apparatus, while control groups received *Haemoproteus*-negative cardinal blood or domestic canary (*Serinus canaria domestica*) blood. *Culex quinquefasciatus* mosquitoes exposed to *Haemoproteus* infected cardinal blood survived significantly fewer days than mosquitoes that ingested *Haemoproteus*-negative cardinal blood. The survival of mosquitoes fed on positive cardinal blood had a median survival time of 18 days post-exposure and the survival of mosquitoes fed on negative cardinal blood exceeded 50% across the 30 day observation period. Additionally, mosquitoes that fed on canary controls survived significantly fewer days than cardinal negative controls, with canary control mosquitoes having a median survival time of 17 days. This study further supports prior observations that *Haemoproteus* parasites can be pathogenic to bird-biting mosquitoes, and suggests that *Haemoproteus* parasites may indirectly suppress the transmission of co-circulating vector-borne pathogens by modulating vector survivorship. Our results also suggest that even in the absence of parasite infection, bloodmeals from different bird species can influence mosquito survivorship.

## Introduction

By definition parasites are organisms that live on or in a host organism at the expense of its host (Price 1977). Parasitism can lead to diverse phenotypic and population-level impacts on their hosts, such as decreased fitness, increased mortality, or behavioral manipulation. For example, *Culex pipiens* survivorship and sand fly biting frequency is modulated by *Plasmodium relictum* and *Leishmania* parasitic infection, respectively, and these changes in vector biology significantly increase the efficiency of pathogen transmission (Beach et al. 1985; Gutiérrez-López et al. 2019; Vézilier et al. 2012). Avian malaria is thought to have a similar effect on mosquito vector feeding behavior, and *Culex pipiens* and *Culex quinquefasciatus* (Say) (Diptera: Culicidae) previously exposed to *Plasmodium* parasites demonstrated increases to mosquito biting frequency and altered host preference in the lab (Cornet et al. 2013, 2019). Identifying factors that can modulate pathogen transmission remains a fundamental goal of epidemiology and disease control.

Vectorial capacity is comprised of any parameters that influence a vector’s ability to transmit pathogens (Kramer and Ciota 2015; Macdonald 1957), and provides a theoretical framework for testing and advancing vector-borne disease control. Arthropod vectors of zoonotic agents of disease are often host generalists and take blood meals from diverse vertebrate species (Cadenas et al. 2007; Hamer et al. 2009). In this context, arthropods are exposed to diverse vertebrate-derived blood factors such as proteins, immunological components and pathogen communities (Boothe et al. 2015; Bottino-Rojas et al. 2015; Hamer et al. 2009; Pakpour et al. 2014). The ingestion of pathogens can impact vectorial capacity by modifying vector survivorship, biting frequency, or competence (Koella et al. 1998; Valkiūnas et al. 2014). This can be seen in *Culiseta melanura* infected with eastern equine encephalitis virus (EEEV), as they have reduced survivorship and fitness (Scott and Lorenz 1998). Such changes in parameters could ultimately influence vector-borne disease outbreak potential (Koella et al. 1998; Miller et al. 1989). Even when an arthropod does not play the role as the biological vector for a pathogen (i.e. mosquitoes and *Haemoproteus* parasites), the ingestion of parasites while blood feeding can influence multiple factors determining vectorial capacity for other pathogens, such as vector survivorship or host feeding activity (Valkiūnas et al. 2014). Therefore, to be able to estimate disease outbreaks and identify the most effective control measures, it is necessary to understand how pathogen ingestion influences components of vectorial capacity.

Mosquitoes in the *Culex pipiens* complex (biotypes and hybrids of *Cx. pipiens* Linnaeus*, Cx. quinquefasciatus* Say) are the most important vectors of mosquito-borne viruses in much of the United States because of their dominant role in West Nile virus (WNV) transmission, and play a primary role in the amplification of WNV in avian hosts due to their ornithophilic feeding behavior (Hamer et al. 2009; Kilpatrick et al. 2006), and also serve as bridge vectors to humans (Hamer et al. 2008; Kilpatrick et al. 2005). While feeding on birds, these mosquitoes commonly ingest a suite of avian pathogens (Boothe et al. 2015; Hamer et al. 2013; Poh et al. 2018), which may affect their vectorial capacity for WNV. One group of pathogens frequently ingested are *Haemoproteus* spp. (Haemosporida: Haemoproteidae), avian haemosporidian parasites similar to avian malaria. Whereas avian malaria parasites (*Plasmodium* spp.) are transmitted by mosquitoes, *Haemoproteus* parasites of the subgenus *Parahaemoproteus* are transmitted by *Culicoides* spp. (Diptera, Ceratopogonidae) biting midges and *Haemoproteus* of the subgenus *Haemoproteus* are transmitted by louse flies (Diptera, Hippoboscidae) (Valkiūnas 2005). It has been reported that *Haemoproteus* decreases the survivorship of blood feeding insects such as *Ochlerotatus cantans*, *Pseudolynchia canariensis*, as well as *Culicoides impunctatus* (Bukauskaite et al. 2016; Martínez-de la Puente et al. 2018; Valkiūnas and Iezhova 2004; Valkiūnas et al. 2014; Waite et al. 2012). Consequently, avian *Haemoproteus* parasites could also change the vectorial capacity of *Culex* mosquitoes for WNV by reducing survivorship. Therefore, the objective of this study is to determine if the ingestion of *Haemoproteus* parasites in avian blood meals reduce *Cx. quinquefasciatus* survivorship.

## Experimental design

### Bird and parasite collection

**Fig. 1:**
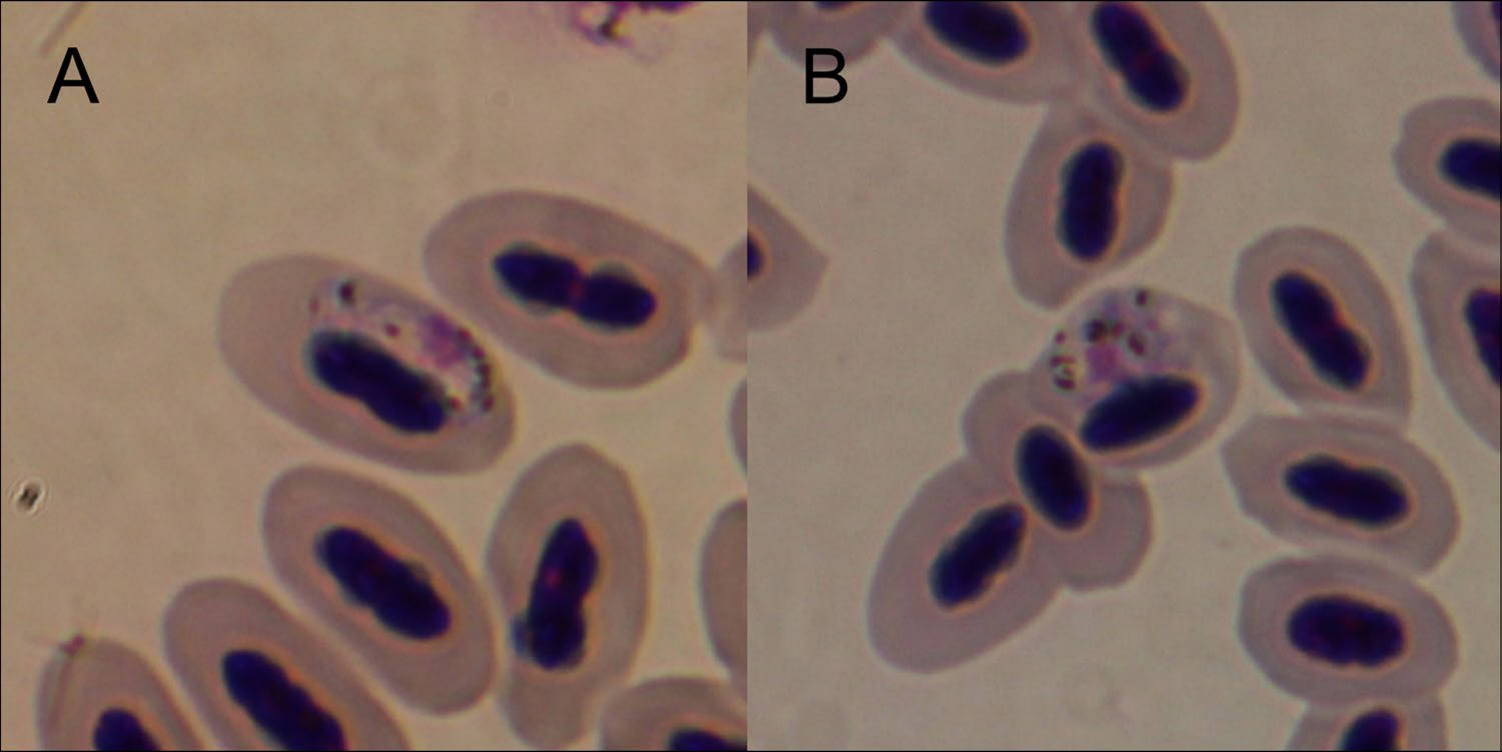
Northern cardinal (*Cardinalis cardinalis*) blood smears with *Haemoproteus* sp. gametocytes present. Two different male Cardinals collected from College Station, TX, USA; Summer 2019

Wild northern cardinals (*Cardinalis cardinalis*) were captured on a residential property in College Station, Texas using 12 m mist nets with 30 mm mesh size (Association of Field Ornithologists, Portland, ME) three times from September–November, 2019. For each captured bird, the species, weight, wing chord, tail length, sex, and age class (hatch year or after hatch year) were recorded (Pyle 1997). We obtained a 200–400 μL blood sample using a 28 ½ gauge syringe by brachial or jugular venipuncture. Prior to bleeding, the syringe was heparinized to prevent blood clotting. In the field, two thin blood smears of about 1–2 μl of blood were air dried, fixed in 100% methanol, and later stained with Giemsa stain (Valkiūnas 2005). The remaining blood was kept on ice and then stored at 4 °C until use. The stained smears were screened in 10 × 10 microscopic fields at 40 × magnification (Fig. 1) to determine the presence of all Haemosporidian parasites (*Haemoproteus* spp., *Plasmodium* spp.*, Leucocytozoon* spp.). Additionally, slides were screened for trypanosomes due to their potential effect on mosquito survivorship. Parasitemia was calculated as the number of parasites per 10,000 erythrocytes, with each microscopic field having approximately 1000 red blood cells. Screening of blood smears was done twice to ensure no false negatives. Birds with a baseline *Haemoproteus* parasitemia of 0.15% were selected for exposure to mosquitoes. Blood considered negative via microscopy was also tested molecularly using PCR for confirmation before feeding to mosquitoes (explained below). All work with birds was approved by the Texas A&M University Institutional Animal Care and Use Committee (IACUC AUP 2018–0144) and Texas Parks and Wildlife Scientific Research Permit (No. SPR-0512–917), and United States Fish and Wildlife Service Migratory Bird Scientific Collecting Permit (No. MB89164A-0).

### Mosquito establishment and care

Wild *Cx. quinquefasciatus* were locally colonized from College Station, Texas in the summer of 2018. Female mosquitoes used for this study were between F12 and F15 generations. Mosquitoes were maintained on a natural day and night light cycle (10 h light, 14 h dark) with a constant 50% humidity at 27 °C. The colony is maintained on commercial whole chicken blood (Hemostat, Dixon, CA) and 10% sucrose solution is provided ad libitum.

### Mosquito feeding through artificial membrane

All mosquitoes used in this experiment were fed using an artificial membrane feeder (Hemotek, Ltd., Blackburn, UK) with 200–400 μL of blood that was heated to 37 °C. In the third trial, blood from two birds with similar parasitemias was pooled together in order to create a large enough bloodmeal to feed mosquitoes artificially. Each cohort was provided with a bloodmeal with a different parasitemia. All blood (cardinals, canaries, etc.) was used within 24 h of collection and blood was provided to mosquitoes for up to 2 h. Parafilm (MilliporeSigma, St. Louis, MO) was used as the blood feeding membrane.

Three cohorts of 100 one week old female *Cx. quinquefasciatus* were offered cardinal blood naturally infected with *Haemoproteus* (N = 124 obtained a blood meal for all three trials combined)*.* After feeding, mosquitoes were immobilized on ice and sorted into three engorgement categories: fully engorged, partially engorged, and unfed. Survivorship was only recorded for fully engorged and partially engorged mosquitoes. One cohort of 100 one week old *Cx. quinquefasciatus* were fed on negative blood samples collected from northern cardinals (N = 31 obtained a blood meal). Only one cohort was presented negative cardinal blood due to difficulty trapping *Haemoproteus*-negative cardinals at this location. To test the effect of bird species on mosquito survivorship, three cohorts of 100 one week old *Cx. quinquefasciatus* were fed blood from domestic canaries (*Serinus canaria domestica*) that were purchased commercially and maintained on campus in an indoor aviary with mosquito proofing (N = 110 obtained a blood meal). Canary blood was heparinized using the same protocol as wild cardinals prior to feeding mosquitoes and also stored at 4 °C until use. Engorged mosquitoes were allowed to feed ad libitum on a 10% sucrose solution until natural mortality or 30 days post-exposure. A maximum of 30 engorged mosquitoes were housed together at once, and the life status (dead/alive) of mosquitoes was recorded daily post-feeding. Dead individuals were removed from cages upon discovery and preserved in 1.5 mL at -20 °C until DNA was extracted.

### Molecular diagnostics and sequence determination

*Haemoproteus* and other Haemosporida (*Plasmodium, Leucocytozoon*) infection and lineage determination was performed on all cardinal and canary blood (including samples with and without parasites evident by microscopy). Additionally, the full bodies of all mosquitoes exposed to *Haemoproteus*-positive cardinal blood had DNA extracted and were PCR tested, as well as a small subset of control mosquitoes exposed to *Haemoproteus*-negative blood. Mosquitoes tested by PCR ranged from 2 to 30 days post-exposure. Infection status in birds was assessed using microscopy primarily, and confirmed using molecular methods. The DNA from 25μL of blood was extracted following the Bio-tek E.Z.N.A. (Omega, Norwalk, CT) manufacturer recommendations with slight modification; blood samples incubated at 70 °C for a minimum of one hour. Whole mosquitoes were extracted using the MagMax CORE Nucleic Acid Purification Kit (Thermofisher, Waltham, MA) following manufacturer recommendations using a KingFisher Flex instrument (Thermofisher, Waltham, MA). Polymerase Chain Reaction (PCR) was utilized to amplify a 478 bp region of Haemosporida cytochrome *b* gene using primers HAEMF and HAEMR2 (Bensch et al. 2000). PCR product (5 μL) was combined with 2μL of ExoSAP-IT (PCR product cleanup reagent, ThermoFisher) to purify the amplicons. Sanger sequencing was performed of both forward and reverse strands (Eton Biosciences Inc., San Diego, CA). Amplification of *Haemoproteus* DNA by PCR was used as evidence of parasite presence in either blood or mosquitoes. To identify which *Haemoproteus* lineage was obtained from PCR samples, a consensus of forward and reverse sequences was aligned, cleaned, and trimmed using Geneious (Biomatters, Inc. San Diego, CA). Clean sequences were queried using GenBank and MalAvi databases to identify similar sequence matches, and mosquito and bird sequences were compared against each other. Sequences from this study were submitted to GenBank and MalAvi with accession number MT762859.

### Data analysis

Survivorship curves and median survival rates (the point at which 50% of mosquitoes died) were created and analyzed using Kaplan–Meier survival estimates (Goel et al. 2010). Due to presence of multiple variables in the experiment (infection status, bird species, engorgement), Cox’s proportional hazards mixed effect model was used (“*coxme”* package) to determine the significance of each variable on the day of mortality post-feeding (Therneau 2015). The full model included Engorgement Size, Bird Species, and Parasite Infection Status, with trial treated as a random effect in all models to control for variation among trials. A minimal model was created with all variables that were significantly associated with mosquito survivorship (Parasite Infection Status, Bird Species)(P < 0.05), and tested using a likelihood ratio test to determine best fit. Means are presented ± standard deviation. Analyses were performed using the R statistical software v3.5.2 (R Foundation for Statistical Computing, Vienna, Austria).

## Results

### The collection of Haemoproteus infected birds

Thirteen northern cardinals were sampled across the three trapping days. Of the 13 birds that were trapped, 11 were positive for *Haemoproteus* infection and no other haemosporidian parasite (*Plasmodium, Leucocytozoon*). Ten of these birds tested positive via microscopy and subsequently PCR. One individual tested positive by PCR only but blood from this individual was not included in experimental infections of mosquitoes. The prevalence of *Haemoproteus* infection in cardinals from trapping at this location was 84.6%, and the prevalence of *Plasmodium* and *Leucocytozoon* was 0%. The parasitemia (% of infected blood cells per 10,000) for all trials had a mean of 0.22% ± 0.077% (range 0.19% to 0.25%). Additionally, all canaries tested negative for Haemosporida.

### Influence of Haemoproteus on mosquito survivorship

**Fig. 2:**
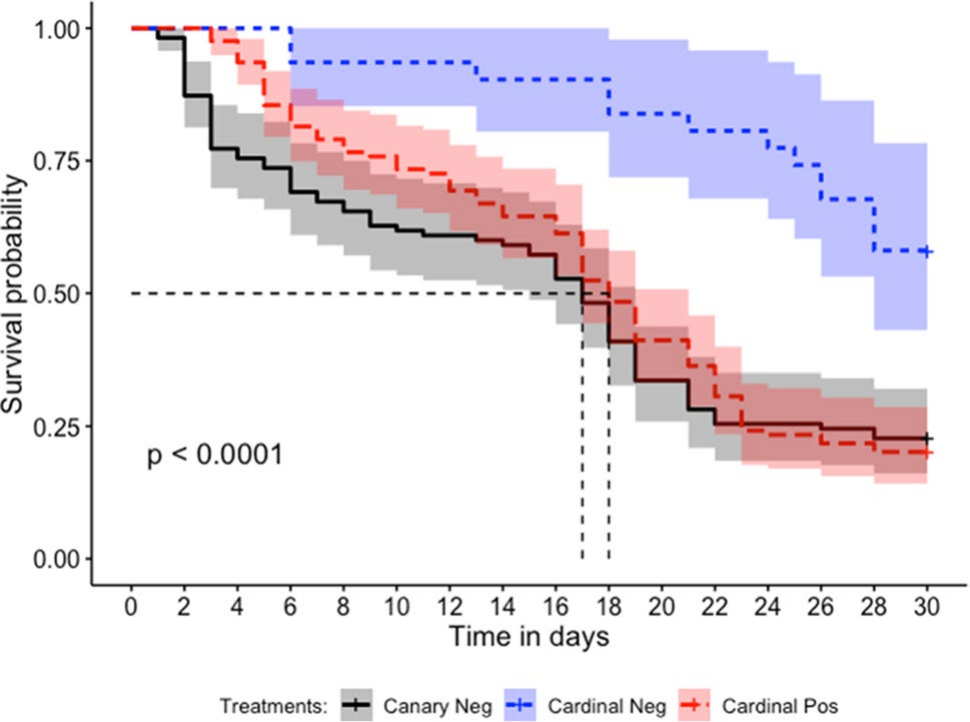
The survivorship curve for *Culex quinquefasciatus* fed on northern cardinal (*Cardinalis cardinalis*) blood containing *Haemoproteus* parasites (red) and without *Haemoproteus* (blue) as well as canary blood with no *Haemoproteus* (gray). Median survival time for mosquitoes fed positive cardinal blood was 18 days and negative canary blood was 17 days. Cardinal negative control fed mosquitoes never reach 50% survival, so their median survival time cannot be calculated. Shaded areas represent standard error of each corresponding color

The Cox’s proportional hazard mixed effects model showed that engorgement (full vs. partial) did not significantly affect mosquito survivorship (X^2 =^ 6.33, df = 2, p = 0.319). Given this result, we combined data from partial (n = 36) and fully engorged (n = 229) mosquitoes to analyze the impact of infection status on mosquito survivorship. When separated by engorgement size, we obtained the same result. Results of the model demonstrate that there was a significant effect of *Haemoproteus* ingestion on mosquito survival (X^2^ = 18.5, df = 1, p < 0.0001; Fig. 2). The median survival time for *Haemoproteus* exposed mosquitoes was 18 days, while survival of the northern cardinal control mosquitoes exceeded 50% across the 30 day experimental period so the median survival time for this group could not be calculated (Fig. 2).

### Influence of bird species on mosquito survivorship

The Cox’s proportional hazard model showed that bird infection status (p = 0.0001) and bird species (p < 0.0001) significantly affected survivorship. The likelihood ratio test included the parameters of infection status and bird species in the best fit model (p < 0.0001, X^2^ = 23.81). When separated by infection status and bird species, a significant difference in survival times was observed with mosquitoes fed on cardinal control blood living significantly longer than those fed on blood from *Haemoproteus* infected and canary control blood (X^2^ = 19.3, df = 2, p < 0.0001). The median survival time for mosquitoes fed on canary control blood was 17 days, one day less than *Haemoproteus* exposed mosquitoes. Calculation of median survival times for mosquitoes exposed to cardinal control blood was not possible as their survival exceeded 50% across the 30 day period (Fig. 2). When trial was included as a random effect, a significant effect was not observed from infection status (p = 0.15) or bird species (p = 0.64). However, trials were not balanced as only one cohort of mosquitoes were fed on negative cardinals while three cohorts were fed on positive cardinals; all three mosquito cohorts fed on positive cardinals had lower survivorship curves than the cohort fed on negative cardinals.

### Haemoproteus DNA in mosquitoes

Thirteen of 124 *Haemoproteus*-exposed mosquitoes tested PCR positive for *Haemoproteus* DNA post-mortem, confirming ingestion of *Haemoproteus* parasites, with the remaining mosquitoes all testing PCR negative. Twelve of the thirteen mosquitoes that tested positive for *Haemoproteus* DNA were fully engorged mosquitoes, with the remaining one being a partially engorged mosquito (Tables 1 and 2). The oldest mosquito that tested positive for *Haemoproteus* DNA was 18 days post-exposure. All positive bird blood and mosquitoes were sequenced resulting in a single sequence that matched 100% identity. The closest lineage in queried databases was *Haemoproteus* (*Parahemoproteus*) sp. (Accession numbers: KY318037, KY318023, KY318019, 100% match), which were previously identified in northern cardinals across the southeastern United States (Walstrom and Outlaw 2017). No similar lineages were found in the MalAvi database.

**Table 1:** Data shown are from all *Culex quinquefasciatus* that took a fully engorged blood meal on *Haemoproteus* positive cardinal blood for each trial. N represents the total number of mosquitoes that died during the time period. Positive mosquitoes are those that were confirmed positive via PCR diagnostics, with the percent infected of total mosquitoes in parenthesis. Days post infection of mosquito death ranged from 0 to 30 and 18 days was the longest that DNA could be detected in mosquitoes

Trial	Days 0–6		Days 7–20		Days 21–30	
	n	pos (%)	n	pos (%)	n	pos (%)
1	11	4 (36)	36	3 (8)	8	0
2	9	2 (22)	7	2 (29)	9	0
3	1	0	2	1 (50)	21	0
Total	21	6 (29)	45	6 (13)	38	0

**Table 2:** Data shown are from all *Culex quinquefasciatus* that took a partially engorged blood meal on *Haemoproteus* positive cardinal blood for each trial. N represents the total number of mosquitoes that died during the time period. Positive mosquitoes are those that were confirmed positive via PCR diagnostics, with the percent infected of total mosquitoes in parenthesis

Trial	Days 0–6		Days 7–20		Days 21–30	
	n	pos (%)	n	pos (%)	n	pos (%)
1	0	0	0	0	0	0
2	2	0	4	0	2	0
3	0	0	1	1 (100)	11	0
Total	2	0	5	1 (20)	13	0

## Discussion

Our results show that the ingestion of a *Haemoproteus* sp. infected bloodmeal is associated with a significant reduction on the survivorship of *Cx. quinquefasciatus* mosquitoes. This study corroborates a prior study documenting reduced survivorship in a Eurasian mosquito (*Ochlerotatus cantans*) after ingestion of *Haemoproteus* infected blood (Valkiūnas et al. 2014). However, this study had a median survivorship for *Cx. quinquefasciatus* of 18 days post-exposure while most *Oc. cantans* died within five days of exposure. *Haemoproteus* spp. are cosmopolitan in birds, but prevalence rates vary by region, time of year, and species of bird ranging up to 100% (Bennett et al. 1978; Chagas et al. 2016; Fecchio et al. 2011; Ishtiaq et al. 2007; Valkiūnas 2005). The prevalence of our study site (84.6%) is very high, and this study site was selected given the awareness of abundant *Culicoides* and previous bird sampling that had documented cardinals with frequent infections with *Haemoproteus* (Martin et al. 2019). *Haemoproteus* parasitemia in wild birds ranges from 0.39% (Fallon et al. 2003) to 6.3% (Valkiūnas et al. 2014) of red blood cells infected, and the average *Haemoproteus* parasitemia of northern cardinals captured in this study (0.22%) is on the lower end of this range. This low parasitemia may be due to chronic, repeated infections by *Culicoides* in the study location. Valkiūnas et al. 2014 demonstrated that mortality was directly related to the parasitemia of consumed blood, which could be an explanation for the difference in the survival length between studies. Mosquitoes in our study were fed on blood with lower parasitemia and had much higher survivorship than those studied by Valkiūnas et al. (2014). Given that these are different species of *Haemoproteus* in different mosquito species, the effect of this parasite may have a differential effect on mosquito survivorship as suggested by prior avian malaria studies (Gutiérrez-López et al. 2020). Additionally, Gutiérrez-López et al. (2019) demonstrates that avian malaria (*Plasmodium*) parasite loads within a mosquito can influence mosquito survival (Gutiérrez-López et al. 2019). Similarly, prior study in another system has also found a dose–response effect of the level of parasite in blood on mosquito survivorship; for example, higher dog heartworm microfilaremia resulted in lower survivorship in *Aedes trivittatus* (Christensen 1978).

Another potential reason for the differences in results obtained between Valkiūnas et al. (2014) and this study is the method of blood feeding of mosquitoes. While Valkiūnas fed directly on birds of high parasitemia, we took blood samples from birds and immediately treated them with heparin, which was then fed to mosquitoes within 24 h after being stored in a 4 °C fridge. Since all treatment types were provided heparinized blood (cardinal positive/negative, canary negative), we would expect to see a similar reduction in survivorship among treatment types by heparin. Storage in the fridge however, may have impacted *Haemoproteus* parasite development, thus introducing a potential artifact into this study. Our studies on *P. relictum* being maintained in captive canaries has stored infected blood in heparin at 4 °C for up to two weeks before passaging to new birds resulting in infections, thus demonstrating parasite viability after fridge storage (Golnar et al. unpublished data). Additionally, the use of a syringe may have damaged parasites in the blood. Ideally, similar studies would feed mosquitoes directly on live parasitemic birds with Institutional Animal Care and Use Committee approval.

Results from our study indicate that *Haemoproteus* parasite was ingested by all mosquitoes that were engorged. Presence of parasite in cardinal blood provided to mosquitoes was initially confirmed by microscopy and PCR, and mosquitoes were PCR tested again postmortem. We found *Haemoproteus* DNA in 13 of 124 mosquitoes that were provided an infectious bloodmeal. A majority of mosquitoes that tested positive for *Haemoproteus* DNA died within 6 days of parasite ingestion, with the remaining positive mosquitoes dying between days 7 and 20. We were able to recover DNA in 30% of mosquitoes that within the first six days post-exposure, which is less than the Valkiūnas et al. (2014) study where 100% of mosquitoes were positive for parasite via dissection and PCR. One explanation for this difference is the differences in survival times. Our mosquitoes survived, on average, 10 days longer than mosquitoes in the Valkiūnas et al. (2014) study. This increased survival time allows for parasite degradation, and more difficulty recovering DNA. Additionally, differences in parasitemia may have affected DNA recovery given that we had up to 29-fold parasitemia difference from Valkiūnas et al. (2014).

Our study also showed that the bird host species influences mosquito survivorship. We report *Haemoproteus*-negative canary control mosquitoes (17 days) had a similarly reduced survivorship as *Haemoproteus-*exposed mosquitoes (18 days). Other studies have also demonstrated the impact of bloodmeal source on multiple aspects of vector biology and vectorial capacity, including survivorship and fecundity (Chikwendu et al. 2019; Noguera et al. 2006; Phasomkusolsil et al. 2013; Shehata 2018). This could be a source of heterogeneity when modeling disease transmission based on mosquito host utilization which varies among regions (Olson et al. 2020). Although we tested the canaries for Haemosporida prior to the experiment and all were confirmed negative, we did not test for other blood parasites that could have influenced mosquito survivorship such as trypanosomes or parasitic nematodes. Additional factors may have contributed to the reduction in survivorship of the mosquitoes by uninfected canaries including differences in captive vs. wild bird nutrition and physiology. For example, captive birds may have a depleted red blood cell count or are overall less nutritious than wild birds. Future research should examine the effect of multiple host species on mosquito survivorship.

One limitation of this study is the low number of mosquitoes fed on control birds (un-infected). This was due to difficulty retrieving *Haemoproteus-*negative northern cardinals from our trapping location. This may have affected the obtained results in multiple ways. Firstly, lower sample sizes can lower the statistical power of our study, which decreases our overall confidence in the results. Because our sample sizes were low for *Haemoproteus-*negative mosquitoes and trials were unbalanced, this introduced the insignificant results from our Cox’s proportional hazards model when trial was included as a random effect. *Additionally*, because this trial utilized one *Haemoproteus*-negative bird, there may have been an individual effect on the results rather than an effect from a lack of *Haemoproteus* parasite. Uninfected birds may have better body condition and health, so blood is more nutritious to mosquitoes and increases survivorship. Alternatively, infected birds have immune compounds (elevated Leucocytes, antibodies, etc.) that negatively impact mosquito survivorship. However, these explanations suggests that *Haemoproteus* impacts mosquito survivorship indirectly, and is detrimental to mosquito survivorship. Future studies should utilize multiple birds for all treatment types to eliminate unbalanced sample sizes.

Our results are based on laboratory observations, but it is likely that *Cx. quinquefaciatus* survivorship is affected by *Haemoproteus* ingestion in nature as well. We previously documented that blood-engorged *Cx. quinquefasciatus* collected in our study site were positive for cardinal DNA 12% of the time (Komar et al. 2018). Furthermore, the *Haemoproteus* sp. lineage used in this study is widespread in northern cardinals across the southern and eastern United States (Walstrom and Outlaw 2017). Additionally, 10 species of *Culicoides* midges, which are the likely vector of *Haemoproteus* infections in cardinals are present in our study location (Martin et al. 2019; Valkiūnas 2005). This includes the ornithophilic *C. crepuscularis*, which has tested positive for Haemosporida DNA (Fallis and Bennett 1961; Martin et al. 2019). Mosquitoes are not known to be vectors of *Haemoproteus* parasites, so parasite DNA amplified from mosquitoes in this experiment may be the result of abortive sporogensis or residual parasite DNA from bloodmeals in the mosquito. This evidence suggests that *Culex* vectors of WNV in the southern United States are frequently exposed to Haemosporida parasites while feeding on birds.

Reductions in *Cx. quinquefasciatus* survival due to *Haemoproteus* exposure may modulate the transmission of vector-borne pathogens such as WNV. For example, WNV has an intrinsic incubation period of between 6–12 days, so the increased mortality of a mosquito fed on a *Haemoproteus*-infected bird makes it less likely to survive long enough to become infectious and transmit virus to a bird or human (Richards et al. 2007; Turell et al. 2001). Additionally, northern cardinals were previously identified as WNV “supersuppressors”, due to their low competency for WNV infection and due to the high utilization by *Cx. quinquefasciatus* in Atlanta, GA (Levine et al. 2016). The current study suggests one more mechanisms whereby cardinals could function as supersuppressors of WNV if they are reducing vector survivorship resulting from *Haemoproteus* infections. On the other hand, *Haemoproteus* and WNV co-infection has unknown consequences on WNV vector competence. Our results suggest that the exposure of mosquitoes to a parasite found commonly in wild birds could influence arboviral transmission, especially given that daily survivorship has a disproportionate impact on vectorial capacity (Kramer and Ciota 2015; Macdonald 1957).

## Conclusions

This study documents *Haemoproteus* parasite ingestion during mosquito bloodmeals is associated with reduced mosquito survivorship. This may have implications on vector-borne disease transmission such as WNV, given that *Cx. quinquefasciatus* vectors frequently feed on birds infected with malaria parasites. Additionally, this study suggests that blood from different species of birds influences mosquito survivorship, introducing another factor to consider in disease systems in which mosquito vectors have broad host associations.
